# Catecholamine-Based Treatment in AD Patients: Expectations and Delusions

**DOI:** 10.3389/fnagi.2015.00067

**Published:** 2015-05-04

**Authors:** Alessandro Stefani, Enrica Olivola, Claudio Liguori, Atticus H. Hainsworth, Valentina Saviozzi, Giacoma Angileri, Vincenza D’Angelo, Salvatore Galati, Mariangela Pierantozzi

**Affiliations:** ^1^Department of System Medicine, Università di Roma Tor Vergata, Rome, Italy; ^2^IRCCS Fondazione Santa Lucia, Rome, Italy; ^3^Policlinico Tor Vergata, Neurofisiopatologia, Rome, Italy; ^4^St George’s University of London, London, UK; ^5^Neurocenter Svizzera Italiana, Lugano, Switzerland

**Keywords:** Alzheimer’s disease, CSF, catecholamine, dopamine, monoaminooxidase inhibitors

## Abstract

In Alzheimer disease, the gap between excellence of diagnostics and efficacy of therapy is wide. Despite sophisticated imaging and biochemical markers, the efficacy of available therapeutic options is limited. Here we examine the possibility that assessment of endogenous catecholamine levels in cerebrospinal fluid (CSF) may fuel new therapeutic strategies. In reviewing the available literature, we consider the effects of levodopa, monoamine oxidase inhibitors, and noradrenaline (NE) modulators, showing disparate results. We present a preliminary assessment of CSF concentrations of dopamine (DA) and NE, determined by HPLC, in a small dementia cohort of either Alzheimer’s disease (AD) or frontotemporal dementia patients, compared to control subjects. Our data reveal detectable levels of DA, NE in CSF, though we found no significant alterations in the dementia population as a whole. AD patients exhibit a small impairment of the DA axis and a larger increase of NE concentration, likely to represent a compensatory mechanism. While waiting for preventive strategies, a pragmatic approach to AD may re-evaluate catecholamine modulation, possibly stratified to dementia subtypes, as part of the therapeutic armamentarium.

## Introduction

In Alzheimer’s disease (AD), efficacious therapies are elusive. Currently approved molecules include acetylcholinesterase inhibitors (AChE-I, such as donepezil, rivastigmine, and galantamine) and the uncompetitive *N*-methyl-d-aspartate (NMDA) glutamate antagonist memantine. These all have demonstrated only weak effects on cognitive functions. Nevertheless, in the absence of evidence-based novel approaches, these drugs remain central to dementia care. To note, recent reports confirm their safe profile, even in Parkinson’s disease (PD) dementia complex (Emre et al., 2014).

In addition, some innovations have been proposed, including newly designed hybrids or alternative routes of administration (Bhavna et al., 2014; Thiratmatrakul et al., 2014). While some authors advocate a more extensive utilization of memantine, definitive data on the glutamatergic transmission involvement in AD are incomplete. Moreover, clinical results have indicated only temporarily limited or disappointing effects of memantine. In an ample series of mild to moderate AD, there were no significant differences in the groups receiving memantine alone or memantine plus alpha tocopherol (Dysken et al., 2014). Thus, the dictum remains that, unfortunately: “the more the disease progresses, the more the ineffectiveness of anti-dementia drugs emerges” (Esposito et al., 2013).

The modern diagnostic paradigm has fueled huge investments in strategies to immunize against the amyloid burden. But, as recently stated, even “BACE inhibitors might reveal as a watershed despite premises and hopes” (Yan and Vassar, 2014); and inhibitors of γ-secretase, including tarenflurbil or semagacestat, “were discontinued due to their lack of cognitive improvement or unacceptable side effects” (Mancuso et al., 2011).

That said, we are witnessing an impressive increase of our diagnostic capability. FDG- and amyloid-PET imaging (Lista et al., 2014) have improved the “early – and differential – diagnosis of dementia” (Perani et al., 2014). Cerebrospinal fluid (CSF) biomarkers that reflect the core pathology of AD (De Souza et al., 2014) appear to facilitate an *etiological* diagnosis even in the prodromal stages of the disorder.

On one hand, an early diagnosis might induce behavior change (e.g., abolition of risk factors, changes in life style). The identification of individuals at risk of developing dementia among people with subjective cognitive complaints or mild cognitive impairment would certainly influences social habits. On the other hand, such a diagnosis will create expectancies that may be difficult to fulfill.

Does the capability “to tackle initial steps of the deranged pathway decades before the clinical explosion” translate into better prognosis? Hampel et al. (2014) claims: “there is a mounting consensus that such disease-modifying compounds and/or interventions are more likely to be effectively administered as early as possible in the cascade of pathogenic processes preceding and underlying the clinical expression of AD.” Well, which molecules became available and were marketed in the last decade?

Several concomitant biochemical alterations appear to be more realistic targets. These include the malfunctioning insulin signaling (Dar et al., 2014; Wirz et al., 2014), the dysfunction of mitochondria-associated membranes, cerebrovascular changes with altered permeability (Diomedi and Stefani, 2014), and disturbed energy metabolism. More radically, some leaders in the functional neurosurgery world are proposing deep brain stimulation of anterior cingulate cortex (or the nucleus basalis of Meynert) (Hardenacke et al., 2013; Laxton and Lozano, 2013). This is not the appropriate context to discuss these *initiatives* (so far, limited to a few patients).

The central hypothesis governing this manuscript is that we might profitably investigate catecholaminergic transmission in AD patients.

It is already established that deficit in endogenous catechol pathways affects cortical plasticity. It was shown, in routine studies with paired pulse protocols, that levodopa and rotigotine might partially revert electrophysiological disturbances attributable to cholinergic deficit. Martorana and Koch recently suggested “the dopaminergic system may well be involved in the occurrence of cognitive decline, often being predictive of rapidly progressive forms of AD” (Koch et al., 2012; Martorana et al., 2013).

These recent statements have returned our attention to catecholamine-mediated functions in aging brain disease. In addition, we are aware of the putative key role of noradrenaline (NE) in shaping the so-called “cognitive reserve” (Robertson, 2013) and the well-known correlation of nigrostriatal impairment with dementia in movement disorders (McKeith et al., 2007; Tatsch and Poepper, 2013). These reflections inspired the current manuscript.

## Catecholamine Therapy in AD

### MAO inhibitors

The therapeutic potential of monoamine oxidase inhibitors (MAOI) in AD has been suggested in light of their neuroprotective properties and augmenting effect on monoaminergic transmission (Klegeris and McGeer, 2000). In the early 90s, Agnoli et al. (1992) performed one of the first pilot studies, followed by several groups (Tolbert and Fuller, 1996; Freedman et al., 1998; Tariot et al., 1998) but results were quite inconsistent (Thomas, 2000).

A promising double-blind, randomized, multicenter trial, for example, provided some encouraging results (Sano et al., 1997, although the baseline score on MMSE was biased).

A 2003 Cochrane review on selegiline and AD (Birks and Flicker, 2003) admitted that, “despite its initial promise, and its role in the treatment of PD sufferers, selegiline for AD has been proved disappointing.” The authors concluded “no evidence of a significant adverse event profile” but also “no evidence of a clinically meaningful benefit for AD” (Birks and Flicker, 2003).

Recent experience is suggesting new potential avenues. We are now aware of the limitations hampering old trials, such as the methodological problems in complex disease like dementia, and the difficulty in interpreting results in the absence of reliable biomarkers (some of which are now available). In other words, it is now possible to pursue trials with: (a) small cohorts (enrolling specific disease subtype, avoiding the bias of non-specific inclusion of mixed dementia type), (b) correlation between biomarkers and cognitive scores – not dominated by mere analogic scoring (such as MMSE scale).

In addition, rasagiline, whose proteiform neuroprotective abilities are undisputed, acquired the potential status of a disease-modifying agent in PD (Hanagasi et al., 2011; Kupershmidt et al., 2012). Recent years are witnessing the possibility that these agents induce protective effects on cognitive performance not merely in PD, but also in normal brain aging.

Not surprisingly, the pipeline of several small companies is recently developing hybrids, combining propargylamine-derived molecules with AChE-I (Zheng et al., 2012; Lu et al., 2013).

This strategy is summarized by Bolea et al. (2013): the paradigm by the Food and Drug Administration-approved drugs based on the “one drug, one target” (donepezil, galantamine, and rivastigmine) might be rephrased into the “one drug, multiple targets” (hence, “a variety of hybrid compounds acting on very diverse targets”) (Schneider et al., 1993; Bolea et al., 2013).

### Levodopa and dopamine agonists

A direct involvement of dopamine in AD was inferred by several pre-clinical, experimental contributions. Himeno presented striking data, showing that the DA agonist apomorphine, in a transgenic murine AD model, not only accelerated amyloid degradation and protected hippocampal neurons from oxidative stress, but also “restored the memory dysfunctions and improved the major pathological hallmarks” (Himeno et al., 2011). In another seminal paper (Kadowaki Horita et al., 2013), bilateral lesions of the dopaminergic input to the prefrontal cortex (PFC) were produced in rats using 6-hydroxydopamine (6-OHDA). Cognitive performance was improved by istradefylline treatment, by increasing dopamine levels in the PFC in both normal and PFC-lesioned rats.

Several different groups have suggested dopamine as a key player in the clinical course of AD (Itoh et al., 1996; Kemppainen et al., 2003; Kumar and Patel, 2007; Mura et al., 2010). Kemppainen et al. examined the DA receptor-binding potential [through PET with the D2/D3 antagonist (11)c FLB 457]; it was found a reduced binding in the right hippocampus, with a positive correlation with verbal memory performance and picture naming (at the Boston Test), supporting studies “to evaluate the efficiency of dopaminergic medication on patients with early AD.”

Mura et al. (2010) stressed the possibility that pathological Abeta oligomers may exert a dysfunctional impact on endogenous catecholaminergic transmission, even before the obvious neurodegenerative structural alterations.

Martorana’s group has highlighted a potential DA deficit (D1-mediated, at least in part) underlying cognitive impairment. More intriguingly, levodopa partially restored cortical transmission (evaluated by TMS tools, Martorana et al., 2008, 2009, 2010, 2011). Levodopa and/or dopamine agonists, such as rotigotine, whose endogenous binding is not limited to D2-like preferring sites, can promote modest but significant cognitive amelioration (Martorana et al., 2013). These findings are not surprising *per se*. A large corpus of pharmacological experiments in 6-OHDA-lesioned rodents demonstrated the efficacy of apomorphine in reverting perturbed behavioral tasks (such as water-maze), thus championing the D2–D1 mixed agonist profile. In our experience with mild PD subjects, levodopa proved effective (or at least without cognitive counterbalance), whilst selective D2 agonists might impair short-term verbal memory, attentional-executive functions, and verbal fluency (Brusa et al., 2003).

### Noradrenaline modulators

Dysfunction of locus coeruleus (LC) is a hallmark of AD (Zarow et al., 2003; Bekar et al., 2012). It was postulated long ago that damage to LC noradrenergic neurons (deposition of neurofibrillary tangles) might contribute to disease progression (Zweig et al., 1989).

Noticeably, NE may participate in the pathogenesis of the amyloid cascade, as suggested in transgenic APP23 mice, when the classical α(1)-adrenoceptor antagonist prazosin reduced the generation of amyloid β in N2a cells, and promoted a cascade of molecular events culminating in benefits to memory function (Katsouri et al., 2013).

Yet, experimental setting has provided conflicting results. For example, 1-month treatment with l-DOPS, NE precursor, improved learning in the Morris water-maze test compared with vehicle-treated mice. l-DOPS increased CNS NA levels, and average latency times in the water-maze test were inversely correlated to NA levels (Kalinin et al., 2012).

In patients, prazosin improved behavioral symptoms in AD patients with agitation/aggression (Wang et al., 2009). Yu et al. (2011), in a population-based study of individuals with incident AD, demonstrated that beta-blockers are also associated with delay of functional decline. In contrast, Gliebus and Lippa (2007) showed that “a trend for worse delayed memory retrieval occurred in patients who were on CNS-active beta-blockers.” Further, beta-adrenergic agonists were anecdotally shown as “memory rescuing” (Gibbs et al., 2010). These inconsistent findings may be attributed to two major biases; (a) inconsistent inclusion criteria and/or (b) concomitant loss of LC functions and ongoing compensatory mechanisms (with variable degree depending on AD stage).

In favor of a strong compensatory mechanism is previous evidence, showing that the increase in NE, following alpha-2 adrenoreceptor blockade in both aging and AD, is detected in absence of metabolite increase (Raskind et al., 1999). This is consistent with partial loss of CNS noradrenergic neurons, with compensatory activation of surviving noradrenergic projections.

A previous elegant work (performed in AD and DLB patients) corroborated this assumption, showing compensation through an increase in tyrosine hydroxylase (TH) mRNA expression in the remaining LC neurons, sprouting of dendrites into the peri-LC dendritic zone, and sprouting of axonal projections to the hippocampus as determined by alpha-2-receptor binding (Szot et al., 2006).

In contrast, some reports (e.g., Fitzgerald, 2010) suggested that, independent of potential loss of LC cells, brain NE levels may be elevated in some persons with AD, representing an etiological factor in some cases of AD, and not merely an epiphenomenon of the disease.

A recent epigenetic study (Mustapic et al., 2013) favors the prevalence of compensatory mechanisms, given that the activity and levels of dopamine beta-hydroxylase was reduced since the early stages AD (advancing the hypothesis for an indication to NA reuptake inhibitors).

## Preliminary Data

Since 2012, we have started to evaluate CSF catecholamine concentrations in dementia patients. Routine lumbar puncture (LP, Stefani et al., 2005, 2006, 2009, 2012; Chiaravalloti et al., 2014; Liguori et al., 2014) was performed in patients during brief admission to our clinic. We also assessed neurocognitive impairment, exclusion of concomitant iatrogenic or metabolic risk factors, and carried out neuroimaging (MRI and/or FDG-PET). Dementia history was <4 years, in line with mild to moderate stages (Table 1 for major epidemiological features).

**Table 1:** **Epidemiological main features of the studied populations (as a whole and split into AD-like vs. FTD-like)**.

	Age (years)	Education (years)	Disease duration (months)	MMSE (score/30)
DET (*n* = 40)	69.80 ± 4.16	7.57 ± 3.54	29.75 ± 16.43	21.18 ± 3.00
AD (*n* = 26)	70.27 ± 4.43	7.31 ± 2.74	28.92 ± 16.07	21.10 ± 3.69
FTD (*n* = 14)	68.93 ± 3.58	8.07 ± 4.78	31.54 ± 18.28	21.34 ± 2.46
*P*	0.21	0.90	0.80	0.94

In this relatively small cohort of patients, we analyzed the CSF concentration of DA and NE [plus some major metabolites of DA and 5-hydroxytriptamine (5-HT) namely homovanillic acid (HVA) and 5-hydroxyindolacetic acid (5-HIAA), previously indicated as possible biomarkers correlate with behavioral disturbances].

An age-matched control cohort was composed of patients with other neurological diseases (mostly radiculopathies) and manifesting no cognitive decline (mean MMSE 29.7, data not shown).

For the meantime, the first analysis revealed non-significant alterations in catecholamine CSF levels; nevertheless, demented patients showed the tendency toward a decreased DA/NE ratio with respect to controls.

In light of patients’ clinical profile ascertained along 2-year follow-up, and after FGD-PET completion and the acquisition of the complete CSF profile (including the assessment of Abeta 1–42, total tau, and phospho-tau proteins), the dementia group was subdivided into: AD-type (*n* = 26) and frontotemporal dementia (FTD) (*n* = 14; see Table 2). Table 2 reflects this more selective analysis.

**Table 2:** **Main CSF results (concentrations are expressed in nanomoles per liter)**.

	DA	NE	HVA	5-HIAA	HVA/5-HIAA
DET (*n* = 40)	0.19 ± 0.24	0.66 ± 0.28	81.63 ± 143.51	43.71 ± 19.13	1.66 ± 3.28

	**DA**	**NE**			

AD (*n* = 26)	0.13 ± 0.12	0.70 ± 0.32			
FTD (*n* = 14)	0.30 ± 0.35	0.56 ± 0.15			
*P*	0.06	0.20			

	**DA**	**NE**			

CTR (*n* = 16)	0.67 ± 1.43	0.49 ± 0.10			
*P* vs. AD	0.10	0.01			
*P* vs. FTD	0.91	0.14			

The tendency for a lower CSF DA concentration was strictly confined to the AD-like subtype. More importantly, the AD group showed a significant increase of NE concentration; conversely, the HVA/5-HIAA ratios, investigated in previous papers as a potential biomarker for behavioral FTD subtype, in our hands revealed no difference among studied groups.

## Discussion and Conclusion

1. This manuscript has tried to shed new lights on catecholamine-based treatment in AD. Asides from the opportunity to modulate dopamine-sensitive signs in patients attributable to LBD (not addressed here), recent data support a renewed interest around the usefulness of dopaminergic agents in handling cortical dementia, particularly AD-type.

This brief literature review imposes an extreme caution. Arguments to build up a therapeutic indication are still poor. After all, levodopa-centered therapy as add-on treatment in a cocktail regimen for AD patients has been tested only in observational clinical studies (or centered on electrophysiological indexes) and in very limited trials.

Our CSF data indicate that AD patients have increased NE levels, relative to aged, neurological controls without dementia. These findings suggest compensatory changes, but do not offer a simple therapeutic answer. In fact, they could be attributed, *per se*, to non-specific processes, representing a mere correlate of senescence (in light of a well-known intrinsic vulnerability of VTA/SN/LC regions in any aging brain).

In addition, a precise pathogenesis role of catecholamine along the course of AD is still questionable. Strong is the evidence, for instance, on a perturbed catecholamine vesicular system shared by different movement disorders, but not playing relevant role in AD (Goldstein et al., 2013).

2. In the short term, as sample size increases, we will assess the relation of CSF pattern to specific clinical phenotypes; in this content, longitudinal assessment may facilitate the understanding of the impact of DA- or NE-based therapy on specific behavioral traits.

Our students recall the clinical presentation of Alois Alzheimer’s patient, D. Frau Auguste, dominated by emotional distress and infidelity/excessive jealousy, before or in combination with cognitive symptoms (Geda et al., 2013). Still nowadays, despite the fact that we gather a clinical diagnosis of “probable AD” *decades* in advance, psychic alterations are prominent and critical, mostly in early-onset AD.

Although disease-modifying therapies are the “Holy Grail” to pursue symptomatic therapies, addressing neuropsychiatric aspects are also important for the quality of life of patients and caregivers (Geerts et al., 2013). Amongst the unmet needs, are the relevance of delusions (not simply attributable to psychotic derangement, Ismail et al., 2011.) and the severity of apathy. Borroni et al. (2010) in reminding that >50% AD patients manifest psychiatric disturbances such as psychosis, depression, agitation, disinhibition, aggression, hyperactivity, and socially intrusive behaviors, consider likely candidates the involvement of DA- or serotonin-related pathways and associated genetic variabilities. Yet, these assumptions do not translate into operative pharmacological strategies. As a matter of fact, the current attitude shared by general practitioners (adding SSRI or SNRI or trazodone) is just empirical (Mizukami et al., 2009; Sepehry et al., 2012).

Particularly elusive is the treatment of apathy. As reviewed by Vilalta-Franch et al. (2013), apathy syndrome in Alzheimer’s disease (ASAD) has a prevalence and incidence/year respectively 21 and 10.6%; it has been reported that ASAD persisted in 61.2% of patients after 1 year, causing an increased functional disability, but no relationship with cognitive impairment or increased caregiver burden was detected. Apathy treatment, despite occasional trial with modafinil or methylphenidate, remains a challenge (Herrmann et al., 2008; Padala et al., 2010; Frakey et al., 2012).

3. A comprehensive review (Schneider et al., 2014), examining late-stage AD drug development from 1984 to 2013, reminds that “the predominant drug targets have been the cholinergic system and the amyloid cascade.” Yet, adjourned “regulatory guidance and oversight have evolved to allow for enrichment of early-stage AD trial samples using biomarkers and phase-specific outcomes.” Hence, although “validated drug targets for AD remain to be developed (and only drugs that affect an aspect of cholinergic function have shown consistent, but modest, clinical effects in late-phase trials), there is opportunity for substantial improvements in drug discovery and clinical development methods.”

It is uncertain whether this hope of catching the disease in its presymptomatic phase (Wirz et al., 2014) is realistic. Of course, we should agree with opinion leader as Hampel when declaring that treatments “*need to be applied before* various molecular mechanisms converge into an irreversible pathway leading to morphological/structural damages” (Hampel et al., 2014). Yet, most modern health systems cannot tolerate decades of diagnostic protocols whose therapeutic effectiveness is unvalidated. The cost of one new drug ranges from an optimistic $2.0 billion to $5.7 billion (95% CI $1.5–2.9 billion) (Scott et al., 2014).

In other words, we doubt that “advances in the study of pre-clinical AD have driven the recognition that efficacy of at least some AD therapies may depend on initiation of treatment before clinical manifestation of disease, leading to a new era of AD prevention research” (Langbaum et al., 2013).

Truly enough, upcoming years will deal with a much more pragmatic vision of AD therapy, which explore treatable impairment such as vascular co-morbidity (Diomedi and Stefani, 2014) or insulin dysfunction (Wirz et al., 2014).

In conclusion, and despite conflicting findings, there is the opportunity to foresee extended and blind trials dedicated to clarify to what extent MAOI, dopamine agonists, levodopa, or NE modulators may act synergically with AChE-I since the first AD stages and/or in selected behavioral phenotypes.

## Conflict of Interest Statement

The authors declare that the research was conducted in the absence of any commercial or financial relationships that could be construed as a potential conflict of interest.
